# Lead Poisoning in a Mother and Her Four Children Using a Traditional Eye Cosmetic — New York City, 2012–2023

**DOI:** 10.15585/mmwr.mm7330a2

**Published:** 2024-08-01

**Authors:** Paromita Hore, Slavenka Sedlar, Jacqueline Ehrlich

**Affiliations:** 1 Bureau of Environmental Disease and Injury Prevention, New York City Department of Health and Mental Hygiene, New York, New York.

**Summary**

**What is already known about this topic?**

Surma, a traditional eye cosmetic, has been recognized as a source of lead exposure among adults and children. Banned in the United States, surma is often purchased abroad and hand-carried into the country.

**What is added by this report?**

A blood lead level above CDC’s blood lead reference value detected in a child in New York City in 2012 was associated with use of surma. Over a period of 11 years, four other family members, including the child’s mother and three younger siblings, were also affected.

**What are the implications for public health practice?**

Cultural practices often persist despite health warnings and can span generations, highlighting challenges in risk communication and the importance of comprehensive family follow-up once a family member at risk of lead exposure is identified.

## Abstract

Even low levels of lead in children’s blood are associated with developmental delays, difficulty learning, and behavioral issues. Adults are also vulnerable to the detrimental health effects of lead exposure. The New York City (NYC) Department of Health and Mental Hygiene receives blood lead test results for NYC residents and conducts investigations of lead poisoning cases. Blood lead testing of a child aged 4 years in 2012 led to the discovery of blood lead levels above the CDC blood lead reference value of 3.5 *μ*g/dL in the child as well as four other family members over a period of 11 years, including the child’s mother and three younger siblings born during 2012–2016. The only potential source of lead exposure identified for all cases was the use of surma, a traditional eye cosmetic, which was found to contain 390,000 ppm lead. The cases in this report highlight the challenges of risk communication when deeply ingrained cultural practices, such as the use of surma, persist despite health warnings. Moreover, they highlight the intergenerational nature of such practices and the need for comprehensive family follow-up once a member is identified as being at risk. These products continue to be available globally, even in places such as the United States where sales are prohibited. Multistakeholder efforts involving local and global engagement could promote reformulation of these products at the countries of origin to eliminate lead as an ingredient.

## Introduction

No safe blood lead levels (BLL) in children have been identified, because even low levels of lead in blood are associated with developmental delays, difficulty learning, and behavioral issues.* Adults are also vulnerable to the detrimental health effects of lead exposure.^†^ Surma, a traditional eye cosmetic (also known as kohl in certain regions of the world), is typically galena- (lead sulfide) based with extremely high levels of lead, and has been recognized as a source of lead exposure among adults and children (*1*–*4*). It is widely used in the Middle East, India, Pakistan, parts of Africa, and increasingly in other parts of the world because of migration (*1*,*2*). The population of New York City (NYC) comprises a wide spectrum of global cultures; associated with this diversity is a range of customs and practices, including the use of cultural products, such as surma. Among some communities, surma serves a variety of cultural purposes. It is used on children as young as newborns in the belief it will protect against misfortune or injury, improve eye health, or to adhere to religious traditions. These practices can continue into adulthood. Surma is typically a fine powder with substantial potential for hand-to-mouth exposure, especially among infants and children. Although banned in the United States, surma is often purchased abroad by South Asian, Middle Eastern, and North- and West-African immigrants and hand-carried into the United States. Despite warnings about potential lead exposures, its use persists within these communities. This report describes BLLs above the CDC blood lead reference value (BLRV) of 3.5 *μ*g/dL among five NYC residents (a mother and her four children) associated with surma use.

## Methods

The NYC Department of Health and Mental Hygiene (DOHMH) receives blood lead test results for NYC children and adults and conducts follow-up investigations of persons with BLLs above CDC’s BLRV^§^ (*5*). During these investigations, DOHMH administers a risk assessment questionnaire and conducts environmental sampling to identify the potential lead sources. DOHMH collects samples of consumer products suspected to contain lead and reportedly mouthed or ingested by the lead-exposed person. Samples are analyzed for lead by an accredited laboratory using the Environmental Protection Agency Method SW6020.^¶^ Laboratory results, along with a description of each sample as conveyed by the family or retrieved from product packaging, such as the product name, origin, and usage information, are stored electronically in a proprietary structured query language (SQL) server database.

This report describes a family of five whose BLLs above CDC’s BLRV were associated with use of surma. The family members included four children born during 2008–2016 and their mother, whose BLL above CDC’s BLRV was identified during one of her pregnancies. Information on BLLs, laboratory results, risk assessments and case coordination notes were retrieved using SQL Server Studio Management (version 18.12.1; Microsoft). The DOHMH Institutional Review Board reviewed the report and determined that the activity did not constitute human subjects research.

## Results

**FIGURE 1:**
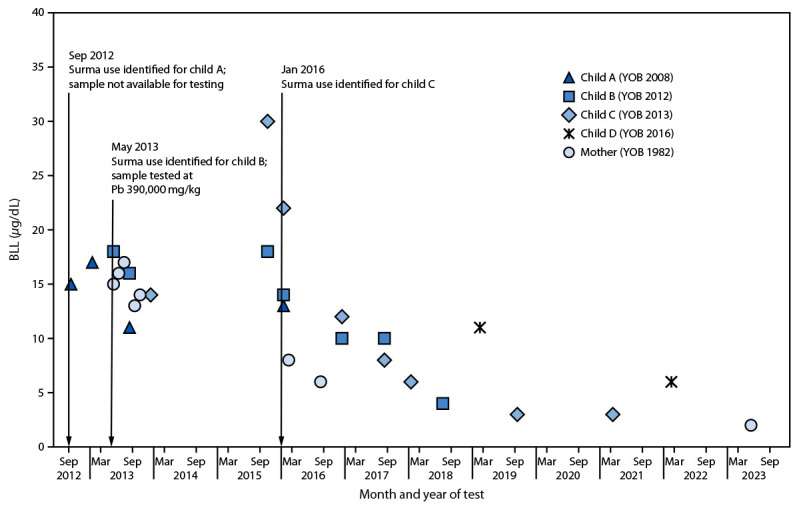
Blood lead levels of a mother and her four children with a history of surma use — New York City, 2012–2023* **Abbreviations:** BLL = blood lead level; DOHMH = Department of Health and Mental Hygiene; Pb = lead; YOB = year of birth. * Before June 2019, Local Law 1 of 2004 required DOHMH to conduct environmental investigations for New York City children with a BLL of ≥15 *μ*g/dL. Since then, DOHMH has been conducting environmental investigations for all children with a BLL of ≥5 *μ*g/dL, and starting in March 2022, DOHMH has been providing these services to all children with a confirmed BLL of ≥3.5 *μ*g/dL.

In September 2012, routine testing detected a BLL of 15 *μ*g/dL in an asymptomatic child, aged 4 years (child A), who had arrived from Pakistan 16 months earlier (Figure 1). DOHMH inspectors visited the child’s home and conducted environmental sampling. None of the 20 paint X-ray fluorescence measurements or 11 dust wipe measurements collected exceeded the regulatory guidelines for paint (1 mg/cm^2^) and dust (40 *μ*g/ft^2^ for floors, 250 *μ*g/ft^2^ for windowsills, and 400 *μ*g/ft^2^ for window troughs) in place at the time. DOHMH conducted a comprehensive risk assessment interview, during which the only potential lead source identified was use of surma on the child; however, a sample was not available for testing. The family was informed about the dangers of lead in surma and advised to discontinue using it. However, 4 months later, a follow-up BLL for child A was 17 *μ*g/dL, indicating no change.

**FIGURE 2:**
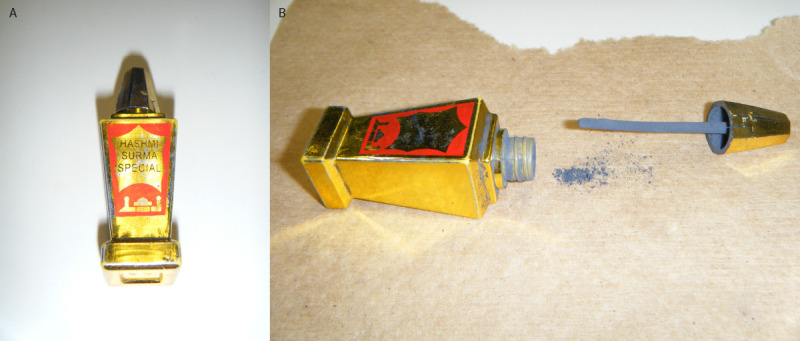
Image of Hashmi Surma Special container (A) and powdered product (B) used by five family members with blood lead levels exceeding CDC’s blood lead reference value — New York City, 2012–2023 Photos/New York City Department of Health and Mental Hygiene

Because of the BLL of 15 *μ*g/dL identified in child A in September 2012, DOHMH nurses recommended testing of child A’s asymptomatic sibling (child B), aged 6 months (born in 2012); however, testing was not conducted until May 2013, when child B was aged 15 months. Child B’s BLL at that time was 18 *μ*g/dL. At approximately the same time, the children’s mother, who was pregnant and asymptomatic, received routine prenatal blood lead testing, and had a BLL of 15 *μ*g/dL. Comprehensive risk assessments for child B and the mother identified surma as the only potential source of lead exposure. A surma sample was obtained, tested, and found to contain 390,000 ppm of lead. The surma (brand name Hashmi Surma Special) was manufactured in Pakistan and hand-carried by the family to NYC (Figure 2). The family was educated again about the potential for lead exposure from surma use and advised, both by DOHMH and the children’s pediatrician, to stop using it. In December 2013, the mother delivered a baby (child C); around the time of delivery, both the mother and newborn had BLLs of 14 *μ*g/dL, which were below DOHMH’s intervention level of 15 *μ*g/dL at that time. In June 2014, their pediatrician reported the mother and three children were in Pakistan.

Approximately 1 year later (October 2015), after returning from Pakistan, child B and child C received retesting. No decline in child B’s BLL was observed (18 *μ*g/dL), and child C’s BLL had increased from 14 *μ*g/dL at birth to 30 *μ*g/dL. During a comprehensive home investigation at the family’s new residence, none of the 132 X-ray fluorescence or 13 dust wipe samples detected lead above the regulatory thresholds. As was the case in 2012, the only potential lead source identified was recent use of surma. A surma sample was not available for testing, but the family was again advised to discontinue use. A few months later, in early 2016, the BLLs for all three children and the mother had declined; child A’s BLL was 13 *μ*g/dL (child A’s last documented BLL), child B’s BLL had declined from 18 to 14 *μ*g/dL, child C’s from 30 to 22 *μ*g/dL, and the mother’s from 14 to 8 *μ*g/dL. Two years later, in 2018, BLLs in child B and child C continued to decline (4 *μ*g/dL and 6 *μ*g/dL, respectively). In 2019, when child C’s BLL was 3 *μ*g/dL, DOHMH identified a fourth child (child D), aged 2 years (born in 2016), with a BLL of 11 *μ*g/dL. Considering the history, surma use was suspected; however, the family reported not using it on this child. By February 2022, 3 years later, child D’s BLL had declined to 6 *μ*g/dL. It had taken approximately 5 years for the BLLs of child B and child C to decline to levels <5 *μ*g/dL. By May 2023, the mother’s BLL had declined to 2 *μ*g/dL. DOHMH continues to monitor the BLLs in this family.

## Discussion

The case series described in this report, highlighting a family’s prolonged exposure to lead through use of surma, illustrates persistent challenges associated with reducing exposure to lead in a multicultural urban setting. Surma and similar lead-sulfide or galena-based traditional eye cosmetics (e.g., kohl and tiro) are used around the world (*1*,*2*,*5*–*7*). DOHMH has investigated numerous cases of children and adults with BLLs above CDC’s BLRV associated with use of surma found to contain lead concentrations as high as 980,000 ppm (*6*). Although potential for lead exposures associated with surma use has been previously documented, this report is unique in describing intergenerational use of a product and culturally embedded nature of its use, as multiple family members were exposed successively, despite health warnings and prevention efforts. The family continued using surma even after being advised repeatedly to discontinue its use, likely prolonging the time for BLLs to decline below CDC’s BLRV. The absence of symptoms among the mother and her children with BLLs above CDC’s BLRV, identified through New York State–mandated blood lead testing (*8*,*9*), underscores the importance of systematic surveillance and follow-up activities to identify sources of lead exposure. However, effectiveness of blood lead surveillance depends on testing mandates, and might miss populations at risk of lead exposure who do not routinely receive testing.

### Limitations

The findings in this report are subject to at least one limitation. Surma samples were not always available for lead testing when suspected as a potential source of exposure based on family members’ self-reports and history. Nonetheless, DOHMH has tested approximately 200 samples of cultural powders, including surma, during 2013–2022 through lead poisoning investigations and store surveys, some of which were found to contain extremely high levels of lead (*6*). These data, coupled with the lead analysis of the one available surma sample collected from the family during the follow-up investigations of the children and their mother, and the corresponding self-reports of surma use, as well as the exclusion of other potential sources based on comprehensive inspections and risk assessment, provide strong support that the likely source of lead exposure in this series of cases was surma.

### Implications for Public Health Practice

The cases in this report highlight the challenges of risk communication when deeply ingrained cultural practices, such as the use of surma, persist despite health warnings, which are available and are disseminated in multiple languages by trusted members of the community. Moreover, they highlight the need for comprehensive family follow-up once a member of the family is identified as being at risk. Travel outside of the United States, as in this case of the family visiting Pakistan, should prompt an investigation of the potential use of traditional consumer products. Persons with cultural connections abroad, and who use products such as surma, might also be more likely to have extended absences from the United States because of travel, making follow-up challenging, and leading to longer intervals between blood lead testing, resulting in ongoing exposures and longer time for BLLs to decline. Although DOHMH has removed thousands of hazardous consumer products from NYC store shelves via local enforcement, these products are often hand-carried from abroad (*10*). These products continue to be available globally, even in places where sales are prohibited, leading to lead exposures worldwide and potentially contributing toward detrimental health outcomes. Long-term multistakeholder efforts involving local and global engagement are needed to reformulate these products in their countries of origin to eliminate lead as an ingredient.

## Footnotes

*
https://www.cdc.gov/lead-prevention/about/index.html

†
https://www.atsdr.cdc.gov/csem/leadtoxicity/who_at_risk.html

§ Before June 2019, Local Law 1 of 2004 required DOHMH to conduct environmental investigations for NYC children with a BLL of ≥15 *μ*g/dL. Since then, DOHMH has been conducting environmental investigations for all children with a BLL of ≥5 *μ*g/dL, and starting in March 2022, DOHMH has been providing these services to all children with a confirmed BLL of ≥3.5 *μ*g/dL.

¶
https://www.epa.gov/sites/default/files/documents/6020.pdf
